# Unusual Findings in Down’s Syndrome: Hand Agenesis and Hypospadias

**DOI:** 10.7759/cureus.32311

**Published:** 2022-12-08

**Authors:** Assaad Kesrouani, Reem Obeid, Linda Daou, Zakhia Saliba, Fadi Sleilaty

**Affiliations:** 1 Obstetrics and Gynecology Department, Saint Joseph University, Beirut, LBN; 2 Pediatrics Department, Saint Joseph University, Beirut, LBN; 3 Plastic and Reconstructive Surgery Department, Saint Joseph University, Beirut, LBN

**Keywords:** ultrasound, prenatal, limb, hypospadias, down syndrome

## Abstract

A baby with Down syndrome presented initial findings at the first-trimester ultrasound of increased nuchal thickness and unilateral hand agenesis. During follow-up, other elements were found mainly hypospadias. This report emphasizes through prenatal and postnatal imaging the phenotypic variability of Down syndrome babies.

## Introduction

Down syndrome can include many phenotypic features and is commonly associated with increased NT and soft signs on the first-trimester ultrasound screening. Heart, face, and renal abnormalities are ultrasound's most frequent abnormal traits; Down syndrome phenotype has been extensively described in the medical literature [1-3]. We report an unusual case of Down syndrome in a 38-year-old patient Gravida 2 Para 0 with non-consanguineous marriage, and no relevant medical or family history. She has a BMI of 27.3 and is of Caucasian ethnicity.

## Case presentation

**Figure 1:**
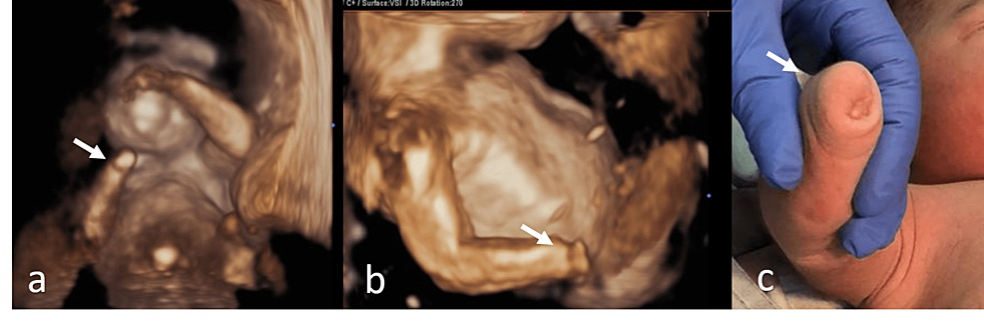
Hand agenesis (white arrow) at 21 weeks (a), 35 weeks (b) and at birth (c)

At 13 weeks gestational age, ultrasound confirmed a viable intrauterine pregnancy with fetal biometry of 12 weeks + 6 days of gestation, along with an increased nuchal translucency (4.9 mm) and a short fetal nasal bone. We suspected a hand agenesis since the hand was not seen clearly. Although invasive testing for karyotype analysis was proposed, the parents refused any prenatal intervention and stated that regardless of the baby’s condition, they will continue the pregnancy. Sonography at 17 weeks+ and 6 days showed a male fetus with adequate biometry. At this time, the diagnosis of hand agenesis was manifest with the absence of an amniotic band, and teratogenic agent exposure. There was also bilateral pyelic enlargement and the heart evaluation was also observed to be within normal limits. The situation was discussed again with the parents who maintained their wish to continue the pregnancy but asked for amniocentesis. It showed a karyotype of a male fetus with trisomy 21. Microarrays analysis was not done because of financial issues. Morphological ultrasound at 21 weeks confirmed prior findings about the hand agenesis (Figures 1a-1c) and revealed the following additional features: atrioventricular canal, pelvic dilation, prefrontal thickening, and Sandal sign.

**Figure 2:**
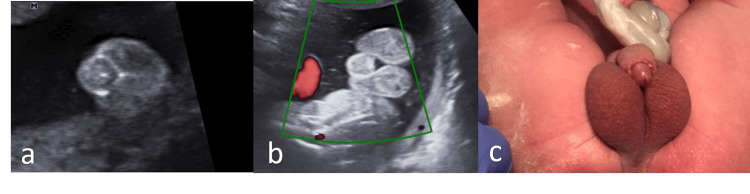
Hypospadias appearance at 21 weeks (a), 35 weeks (b) and at birth (c)

The vermis appeared normal on this exam whereas the genitalia showed an abnormal appearance in favor of hypospadias (Figures 2a-2c).

Following spontaneous labor, a 3.1-kg baby was delivered at 39 weeks by cesarean section for a pathological fetal heart rate with an Apgar score of 10 at one and five minutes. The baby was discharged on day 2 and was followed by a pediatrician. The patient’s written consent was then obtained for the scientific use of the data and images. The study has been approved and registered by our institution’s review board (CEHDF-1762).

## Discussion

Down syndrome is the most common human chromosomal disorder and a leading cause of intellectual disability and birth defects [1]. The major congenital anomalies occurring in infants and fetuses are cardiac anomalies (44%), followed by digestive system anomalies (6%), musculoskeletal system anomalies (5%), urinary system anomalies (4%), respiratory system anomalies (2%), and other systems anomalies (3.6%) [1]. Some malformations, specifically cardiac anomalies, increase the risk of mortality In Down syndrome babies [3]. The skeletal finding characteristics of Down syndrome are short hands, feet, and digits, a short curved fifth finger (dysplasia and shortening of the mid-phalanx) or clinodactyly of the fifth finger with a single flexion crease. Some studies also report a limb reduction in 14% of the musculoskeletal system anomalies [1,2]. Defect prevalence seems to differ by infant sex more than by maternal age, with limb deficiencies being more common in males as compared to females [2]. Terminal transverse defects of limbs refer to the absence or hypoplasia of distal structures of the limb that are relatively normal proximal structures. Most of these cases occur sporadically and primarily involve the upper limb. The majority of these malformations are considered to be non-genetic and could be related to environmental causes [4]. Amniotic band syndrome, teratogen exposure, and vascular accidents can lead to Amelia [5]. Chorionic villus sampling has also been associated with amputation of an extremity and should not be performed before 11 weeks because earlier CVS is associated with fetal transverse limb abnormalities. [6]. In our case, chorionic villus sampling was not performed, and the hand abnormality was noted before performing the amniocentesis. While some studies list hypospadias as related to trisomy 21, the majority of authors consider it a rare finding [1,2,7]. A recent report of VACTERL association (including vertebral, anal, cardiac, tracheoesophageal, renal, and limb abnormalities) in a baby with trisomy 21 emphasizes the need for a VACTERL workup when diagnosing a limb defect in suspected cases of trisomy 21 [8]. In such cases, a microarray analysis could possibly help to diagnose anomalies associated with Down syndrome.

## Conclusions

The association of hand agenesis and hypospadias in Down syndrome cases has not been reported in the medical literature. These unusual features should prove once again the extent to which actual Down syndrome cases can differ from textbook characteristics. Although rare, limb abnormality or hypospadias may be associated with Down syndrome, and this should be discussed with the parents upon finding an increased NT with rare morphological features.
